# The Harm of Metabolically Healthy Obese and the Effect of Exercise on Their Health Promotion

**DOI:** 10.3389/fphys.2022.924649

**Published:** 2022-07-14

**Authors:** Liqiang Su, Yihe Pan, Haichun Chen

**Affiliations:** ^1^ Physical Education of College, Jiangxi Normal University, Nanchang, China; ^2^ School of Physical Education and Sport Science, Fujian Normal University, Fuzhou, China

**Keywords:** metabolically healthy Obese, health Evaluation, metabolic Characteristics, disease Risk, exercise

## Abstract

Obesity and obesity-related diseases [type 2 diabetes, cardiovascular disease (CVD), and cancer] are becoming more common, which is a major public health concern. Metabolically healthy obesity (MHO) has become a type of obesity, accounting for a large proportion of obese people. MHO is still harmful to health. It was discovered that MHO screening criteria could not well reflect health hazards, whereas visceral fat, adiponectin pathway, oxidative stress, chronic inflammation, and histological indicators at the microlevel could clearly distinguish MHO from health control, and the biological pathways involved in these micro indicators were related to MHO pathogenesis. This review reveals that MHO’s micro metabolic abnormality is the initial cause of the increase of disease risk in the future. Exploring the biological pathway of MHO is important in order to develop an effective mechanism-based preventive and treatment intervention strategy. Exercise can correct the abnormal micro metabolic pathway of MHO, regulate metabolic homeostasis, and enhance metabolic flexibility. It is a supplementary or possible alternative to the traditional healthcare prevention/treatment strategy as well as an important strategy for reducing MHO-related health hazards.

## Introduction

Obesity involves an excessive amount of body fat which is harmful to health (Sperrin et al., 2014; van der Klaauw and Farooqi, 2015). Health hazards are often evaluated by disease risk factors. Internal disease risk factors are mostly influenced by the body’s metabolic characteristics. In 1982, scholars advocated using blood lipid and lipoprotein to evaluate metabolic characteristics, and classified those with normal metabolic characteristics who had met the BMI standard of obesity as “metabolically healthy obesity” (MHO) (Herrmann et al., 1982). At present, MHO has become a type of obesity, accounting for a large proportion of obese people. This concept is widely used in research (Mathew et al., 2016). Although MHO is referred to as metabolically normal obesity, the dangers it poses to one’s health should not be underestimated. A large number of studies have found that the risk of cardiovascular diseases (CVDs) in MHO will increase significantly in the future (Fingeret et al., 2018; Li et al., 2019). The metabolic characteristics of MHO are thought to differ from those of healthy people with standard weight, but these subtle metabolic differences cannot be identified through MHO’s inclusion criteria, and the related diseases cannot be well predicted. Therefore, this review argues that the abnormal characteristics of MHO micro metabolism are the causes of an increase in long-term disease risk, examines the mechanism of abnormal MHO micro metabolism endangering health. Additional, current research shows that weight loss medications, but not behavior-based interventions, were associated with higher rates of harms. It is of practical importance to identify an appropriate method, especially no-drug method, of improving the health of MHO, exercise is often considered an effective way to reduce risk factors of obesity related diseases, this review focuses on the characteristics of micro metabolism to explore how exercise can promote MHO health.

## Survey Methodology

A literature search was conducted of PubMed, MedLine, and Web of Science using the search terms “Metabolically Healthy Obese” and “Exercise.” The language is limited to English, species is limited to human. A total of 10,703 articles were searched from each database. Models categorized by their Metabolic Characteristics and representative articles were chosen for inclusion, older literature reviews on the same topic were consulted to ensure key topics were not missed, articles unrelated to the subject of this study were excluded. Finally, 10,604 articles were excluded and 99 articles were included.

## Metabolically Healthy Obesity Screening Criteria and Health Hazards

### Metabolically Healthy Obesity Screening Criteria

The concept of MHO is based on “metabolic health.” Blood lipid and lipoprotein indicators are used to assess metabolic health, as proposed in 1982 (Herrmann et al., 1982). Scholars have developed several screening standards for MHO based on different purposes as research has progressed. Although MHO screening criteria varied, they are inseparable from insulin resistance, metabolic syndrome criteria, or a combination of the two (Durward et al., 2012; Phillips, 2013; Bluher and Schwarz, 2014). MHO has become a type of obesity with certain characteristics. This paper summarizes the MHO adult screening criteria (Table 1). Based on the screening criteria, obesity is classified as MHO and metabolic obesity (individuals exceeding the criteria in Table 1 are metabolic obesity).

**TABLE 1:** Definitions of MHO in adult.

Type	Criterion
HOMA-IR	Meet the obesity standard, and the blood lipid, blood glucose, and blood pressure are normal, HOMA-IR < 2.5, diagnosed as MHO
ATP-Ⅲ	No more than two of the following items are identified as MHO Fasting glucose ≥5.6 mmol/L (or diabetes medications) Systolic BP ≥130 mmHg or diastolic BP ≥85 mmHg (or antihypertensive medications) Triglycerides ≥1.7 mmol/L (or cholesterol-lowering medications) HDL-C <1.04 mmol/L (males), <1.30 mmol/L (females) Waist circumference >102 cm (males), >88 cm (females)
Combined	No more than one of the following items are identified as MHO HOMA-IR ≥1.95 (or diabetes medications) Triglycerides ≥1.7 mmol/L (or cholesterol-lowering medications) HDL-C <1.04 mmol/L (males), <1.30 mmol/L (females) LDL-C ≥2.6 mmol/L Total cholesterol ≥5.2 mmol/L (or cholesterol-lowering medications)

HOMA-IR, homeostasis model assessment-insulin resistance; ATP-III, Adult Treatment Panel-III; BP, blood pressure; HDL-C, high-density lipoprotein cholesterol; LDL-C, low-density lipoprotein cholesterol.

### Health Hazards of Metabolically Healthy Obesity

The primary determinants affecting health and an important basis for measuring MHO’s health are CVD and the risk of all-cause death. MHO has a high level of insulin sensitivity and favorable lipid characteristics, which appears to “protect” the body from developing a metabolic disorder (Sims, 2001; Karelis et al., 2004; Karelis, 2008). Theoretically, patients who meet this profile may have a lower risk of CVD. MHO has a lower CVD risk than obese people with metabolic abnormalities, according to studies (James. et al., 2006; Calori et al., 2011). However, when compared with healthy people with normal weight metabolism, MHO had a higher risk of CVD and death (Caleyachetty et al., 2017; Xu et al., 2018). It shows that people with MHO have more health risks than those who have normal weight metabolism. Based on the “dynamic” and “static” correlation theory, the static metabolic characteristics are thought to be related to the dynamic change of disease risk on the time axis. After excluding interference factors, the static metabolic characteristics of MHO may be the initial cause of dynamic disease risk change. Currently, MHO screening standards cannot show its unique metabolic characteristics, whereas microlevel indicators can deeply and comprehensively reflect the body’s metabolic characteristics. Exploring the relationship between micro metabolic indexes and disease occurrence will help researchers better understand how MHO endangers people’s health.

## The Micro Metabolic Characteristics of Metabolically Healthy Obesity Can Be Harmful to Health

MHO screening criteria is based on insulin resistance, metabolic syndrome, combination of the two. Screening criteria are closely related to metabolic characteristics, at present, the research on the metabolic characteristics of MHO is also developing, the screening of MHO clinical markers and pathogenic factors in the clinical and scientific environment not only helps to accurately identify the metabolic characteristics of MHO but also helps to investigate the causes of MHO harm to the body. The research on MHO has gone deep into lipid metabolism, oxidative stress, chronic inflammation, and metabonomics. In order to distinguish MHO screening criteria (insulin resistance, metabolic syndrome, and combination of the two), this section proposes micro metabolic characteristics to assess MHO metabolic characteristics. Micro metabolic characteristics refer to the metabolic characteristics at the level of lipid metabolism, oxidative stress, chronic inflammation, metabonomics, and clinical molecular level environment. By analyzing the relationship between micro metabolic characteristics and diseases, it is discussed that the micro metabolic characteristics of MHO are the micro basis of endangering health.

### Visceral Fat Endangers Metabolically Healthy Obesity Health

Excessive visceral fat accumulation is a primary risk factor for metabolically unhealthy obesity and related diseases. Visceral fat is frequently used to predict the risk of CVD and other diseases (Du et al., 2015). According to research, visceral fat is an essential factor in determining the risk of MHO in teenagers of standard weight in the future (Remor et al., 2019). Furthermore, it was found that visceral fat of MHO population was increased (Du et al., 2015). It can be seen that an increase in visceral fat is a manifestation of MHO.

Visceral fat is an important immune site, which contains a variety of innate and adaptive immune cells and plays a direct role in immune surveillance and host defense. The increase of visceral fat has a direct impact on the regulation of immune function (Macdougall et al., 2018). Visceral fat’s high pathogenicity is related to its stronger secretion of proinflammatory cytokines, and the biological effects of proinflammatory cytokines are the potential mechanism of increasing the risk of cardiac metabolism. In addition, an increase in visceral fat causes restriction of fatty acid absorption by adipose tissue as well as ectopic fat deposition. Ectopic fat deposition is the direct cause of atherosclerosis, fatty liver, and other diseases (Abd el-Hafez et al., 2014); other detailed studies have shown that increased visceral fat is a marker of increased ectopic fat in other sites, such as the liver and the heart; therefore, abdominal fat distribution can now be considered a marker of ectopic fat in many sites (Smith, 2015). More visceral fat is shown to be harmful to MHO health by secreting proinflammatory cytokines and increasing ectopic fat deposition.

### Adiponectin Reduction Endangers Metabolically Healthy Obesity Health

Adiponectin is a key regulator of metabolic pathway. Individuals with MHO had lower adiponectin levels than those who have normal metabolism (Ding et al., 2018). Hypoadiponectin is a risk factor for atherosclerosis and type 2 diabetes because it promotes the pathological reaction and insulin resistance of the cardiovascular system (Jamar et al., 2017). In MHO, low adiponectin level is an important way to endanger health (Martinez-Larrad et al., 2014; Boyarinova et al., 2016).

The action pathway of adiponectin is related to its receptor. Adiponectin and its receptor 1, when combined, can activate AMPK, which can regulate fatty acid oxidation and improve insulin sensitivity. Adiponectin receptor 2 pathway leads to PPAR *a* ligand synthesis increased with PPAR *a* pathway, which can regulate glucose and lipid metabolism and reduce oxidative stress. Signal transduction of adiponectin is related to the increase of ceramidase activity. Sphingosine 1-phosphate (S1P), which is a diverse second messenger with anti-inflammatory and anti-apoptotic functions, can be produced through ceramide breakdown (Woodward et al., 2017). Through its signal transduction pathway, adiponectin aids in insulin sensitivity, oxidative stress reduction, and inflammation reduction.

Low adiponectin levels in individuals with MHO can endanger health through the reverse process of the above receptor pathway. If adiponectin is employed as a target, increasing its level can reduce the harm. Currently, several drugs used to treat type 2 diabetes and CVD (thiazolidine two ketones and angiotensin receptor blockers) can increase circulating adiponectin levels improving the health benefits (Polyzos and Mantzoros, 2016). It is shown that low adiponectin level is the cause of endangering MHO health, and increasing circulating adiponectin level has become an important way to reduce the harm.

### Increased Oxidative Stress Can Cause Abnormal Metabolically Healthy Obesity Metabolism

Compared with different populations (Standard body weight people with Normal metabolism, Metabolic obesity, Type 2 diabetes with Obesity), it can reflect the characteristics of oxidative stress of MHO. Table 2 summarizes the comparison of oxidative stress levels among standard weight, metabolic health, metabolic obesity, and type 2 diabetes with obesity. In terms of indicators of oxidative stress, they primarily include indicators of oxidative stress degree (ROS, such as TBARS, 8-epi-pgf2a, mitochondrial membrane potential, etc.), antioxidant indicators (glutathione, antioxidant enzymes, total antioxidant status, etc.), and indicators of oxidative stress mechanism (autophagy markers, telomere length, etc.). Judging from the trend of different population indicators, the degree of oxidative stress is small to large in turn, that is, standard weight, metabolic health, MHO, metabolic abnormalities, obesity, and type 2 diabetes. MHO showed abnormalities in TBARS, ox LDL and mtROS, which indicated mitochondrial dysfunction, despite not showing abnormalities in most oxidative stress indicators when compared with those with normal standard body weight metabolism (Zhang et al., 2016; Kruse et al., 2017). Mitochondrial dysfunction is an important pathway of MHO leading to disease (Hu and Liu, 2011; Chattopadhyay et al., 2015). It is shown that MHO undergone small-scale subtle changes at the level of oxidative stress, which may be a part of HMO metabolic micro disorder. This metabolic micro change is thought to increase the likelihood of metabolic abnormalities in MHO in the future.

**TABLE 2:** Comparison of MHO and oxidative stress indexes in different groups.

Literature	Standard body weight people with normal metabolism	MHO	Metabolic obesity	Type 2 diabetes with obesity	Oxidative stress index	Vs.1	Vs.2	Vs.3
Banuls et al. (2017)		N: 29 Age: 42.9 ± 11.2 BMI: 36.6 ± 6.5	N: 53 Age: 44.0 ± 8.1 BMI: 44.3 ± 6.6	N: 31 Age: 51.0 ± 10.4 BMI: 43.5 ± 6.7	t-ROS		↑	↑
					mtROS		↑	↑
					glutathione		=	↓
					Membrane potential		=	↑
					Catalase activity		=	↓
					Superoxide dismutase		=	↓
					Total antioxidant status		=	=
Lwow et al. (2011)	N: 73 Age: 54.4 ± 2.6 MI: 22.4 ± 1.6	N: 27 Age: 56.2 ± 3.6 BMI: 32.3 ± 2.0	N: 61 Age: 55.8 ± 2.4 BMI: 33.8 ± 3.0		AS	=	↓	
					TBARS	↓	↑	
Kim et al. (2019)		N: 34 Age: 35.7 ± 1.62 BMI: 27.0 ± 0.20	N: 34 Age: 37.3 ± 1.41 BMI: 27.1 ± 0.22		8-epi-PGF2a		↑	
Montaigne et al. (2014)		N: 10 Age: 54–76 BMI: >30		N: 10 Age: 57–77 BMI: >30	ROS			↑
					MnSOD			↑
					Cu-ZnSOD			=
					Catalase activity			↑
Kim et al. (2013)	N: 958 BMI: 22.3 ± 0.05	N: 319 BMI: 26.8 ± 0.09	N: 273 BMI: 27.3 ± 0.10		8-epi-PGF2a	=	↑	
					ox-LDL	↓	↑	
Bhansali et al. (2017)	N: 20 Age: 38.9 ± 7.2 BMI: 21.2 ± 1.6	N: 20 Age: 41.5 ± 6.5 BMI: 28.5 ± 2.4		N: 20 Age: 43.7 ± 10.8 BMI: 9.4 ± 3.5	mtROS	↓		↑
					MMP	=		↑
					Autophagy markers	=		↓
Iglesias Molli et al. (2017a)	N: 196 Age: 42 ± 15 BMI: 24.5 ± 3.0	N: 42 Age: 44 ± 15 BMI: 35.3 ± 6.7	N: 102 Age: 51 ± 14 BMI: 36.9 ± 6.2		Absolute telomere length	=	↓	

Vs.1, MHO, vs. Standard body weight people with Normal metabolism; Vs.2, MHO, vs. metabolic obesity; Vs.3, MHO, vs. type 2 diabetes with obesity. ↑, higher than MHO; =, no difference with MHO; ↓, lower than MHO.

### Chronic Inflammatory State of Metabolically Healthy Obesity Is Harmful to Health

MHO population showed that chronic inflammation (Lin et al., 2017), C-reactive protein (CRP), and high-sensitivity C-reactive protein (hsCRP) are nonspecific inflammatory markers that directly participate in the inflammatory response and can be used to evaluate the inflammatory state of MHO. Based on the study, there was no significant change in hsCRP in MHO group compared with those who have metabolic obesity (Poelkens et al., 2014; Pelascini et al., 2016). According to different research findings, the hsCRP content in MHO is also significantly lower than that in obese patients with metabolic abnormalities (Phillips and Perry, 2013; Perreault et al., 2014). Overall, the inflammatory level of MHO was not higher than that of obese patients with metabolic abnormalities. However, when compared with the normal body weight metabolism group, MHO had a higher hsCRP concentration (Perreault et al., 2014; Iglesias Molli et al., 2017b). In addition, van Wijk et al. (2016) found that the risk ratio of coronary heart disease in MHO group with CRP level >2 mg/L was higher than that in MHO group with CRP <2 mg/L. High CRP concentrations were found to be associated with an increased risk of disease in these studies.

CRP is not only related to inflammatory response and immune function but is also directly involved in the occurrence and development of CVD such as atherosclerosis, which is also the strongest predictor and risk factor of CVD (Dong et al., 2019). Therefore, higher concentration of CRP is one of the major factors endangering the health of MHO. It is worth noting that while CRP can be a useful index for determining the inflammatory level of MHO, it can only reflect the inflammatory level of the body from a single perspective. To fully comprehend the inflammatory level of MHO, it must be combined with other indicators for comprehensive evaluation.

### Omics Changes and Health Hazards of Metabolically Healthy Obesity

With the development of various omics technologies, the research on MHO has also entered the omics stage. High throughput omics methods and technologies can test and identify the biological information at the gene, transcription, and metabolism levels of MHO population. The use of omics resources can help researchers better understand the metabolic characteristics of MHO and the sources of its health risks.

Metabonomics studies have discovered that MHO and standard weight normal metabolism, and abnormal metabolism obesity have different metabolic profiles. Some studies have screened out differential metabolites. The metabolic pathway primarily involves the synthesis and decomposition of sugar and fat, and researchers believe that differential metabolites can better reflect the abnormalities of MHO metabolic micro state (Perreault et al., 2014; Park et al., 2015; Rodriguez-Garcia et al., 2017; Almanza-Aguilera et al., 2018; Leal-Witt et al., 2018; Ulaszewska et al., 2019). There are many inducements for the change of micro metabolites of MHO. According to genomic studies, many genes involved in adipogenesis, insulin signal transduction, and insulin resistance pathway are related to the increase of obesity (Scott et al., 2014; Lu et al., 2016). Genes associated with increased obesity are frequently associated with disease risk factors, such as the gene polymorphism of insulin receptor substrate 1 (IRS1), which is linked to the risk of metabolic diseases (Kilpelainen et al., 2011). The genome must be transcribed and translated into bioactive proteins to play its biological role. Therefore, the changes at the transcriptional level are the continuation of changes in the genome. Based on the transcriptomic studies, there were some differences in eIF2, eIF4/P70S6K, and mTOR signals between MHO and metabolic obesity at the transcriptional level (Gaye et al., 2018). mTOR is a key metabolic regulatory factor, which is influenced by growth factors, insulin, fatty acids, amino acids, glucose, and other factors. At the same time, mTOR pathway is also an essential way to realize cell growth and cell cycle regulation. It is shown that the changes in MHO-related metabolic genes lead to the transcriptional changes of cell growth and metabolic regulation, and then to metabolic abnormalities in small molecular metabolites. The abnormality of these metabolites can reflect the metabolic characteristics of MHO, which poses a potential health risk.

**FIGURE 1:**
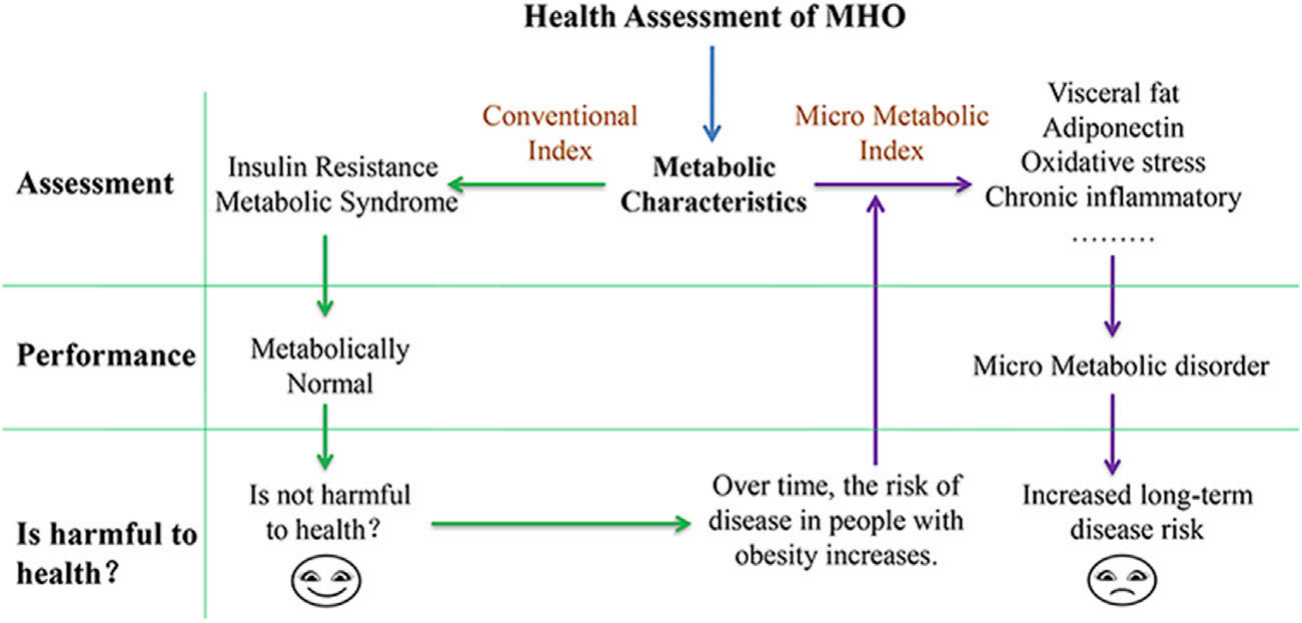
Health evaluation and hazard reason of MHO.

In conclusion, although factors such as heredity, lifestyle, exercise, and emotion influence the metabolic state of the body, when these factors are excluded or controlled, the initial metabolic state of the body may be the initial cause affecting the occurrence of diseases in the future. As shown in the above discussion, despite the fact that MHO screening criteria cannot reflect their own metabolic characteristics, many micro metabolic indexes do show abnormalities, and the metabolic pathways of these micro indexes are often associated with disease pathways. Increased visceral fat, for example, causes the deposition of ectopic fat in blood vessels, which is harmful to blood vessels. Proinflammatory cytokines promote chronic inflammation and damage immune function. Low adiponectin produces an insulin effect that alters glucose and lipid metabolism, causing mitochondrial damage and increased oxidative stress. At the metabolic level, there is an abnormal content of small molecule metabolites, indicating the disorder of micro metabolism. Therefore, this review proposes that MHO is in the state of “micro metabolic disorder.” Although this series of micro metabolic disorders did not cause the indexes of blood lipid and insulin resistance to exceed the standard range, when the body is stimulated by the same internal and external environment, compared with the healthy control, due to the abnormal micro metabolism of MHO, the body’s ability to regulate balance or compensate metabolism becomes smaller, which is more likely to destroy the metabolic homeostasis, which can lead to disease in the long run. Therefore, this abnormal micro metabolism is the reason for MHO’s increased long-term disease risk (Figure 1).

## Ways to Reduce Metabolically Healthy Obesity Health Hazards by Exercise

Exercise is an Important Strategy to Improve MHO Health. Although the micro metabolic state of MHO is not completely healthy, if the current metabolic state can be controlled, disease risk can be greatly reduced or even avoided. Therefore, the research on the means and mechanism of health promotion is beneficial to the development of MHO to a healthy state. In view of the guiding ideology of “preventive treatment,” “disease prevention guide,” “healthy China,” and “precision medicine,” improving modifiable risk factors is seen to be the first choice to reduce the occurrence of MHO diseases. Unhealthy lifestyle can be modified, and it is also a significant risk factor endangering the health of MHO. A large number of studies have used to optimize and improve unhealthy lifestyle to intervene MHO, with positive results (Shin et al., 2017; Rodriguez-Garcia et al., 2018; Xu et al., 2019). Looking at the content of optimizing lifestyle, it is easy to see how sports play a big part of it. Lack of physical activity has become a major risk factor for MHO progressing to metabolic obesity (Goday et al., 2016; De Lorenzo et al., 2017). Less physical activity is associated with higher incidence rate/mortality of MHO (Ortega et al., 2018), whereas increasing physical activity and regular physical exercise can reduce the risk of MHO disease (Arsenault et al., 2009; Janiszewski and Ross, 2010; Ortega et al., 2013; Dalleck et al., 2014). Exercise has been proven to be a supplement or possible alternative to the traditional healthcare prevention/treatment strategy (DiMenna and Arad, 2018). It is also an important strategy to improve MHO health. As mentioned earlier, the abnormal micro metabolic index of MHO is the reason for the increase of disease risk. Is exercise related to the change of micro metabolic environment?

The following sections will look at how exercise can help to reduce MHO health risks in terms of fat distribution, adiponectin, oxidative stress, and chronic inflammation.

### Exercise Reduces Visceral Fat and Improves Metabolically Healthy Obesity Metabolism

Visceral adipose tissue is harmful to metabolic health (Justice et al., 2019) because visceral fat increases the secretion of proinflammatory factors, ectopic fat deposition, and fatty acid oxidation. These changes lead to the decline of metabolic activity, increasing the risk of disease (Goodpaster and Sparks, 2017). Reducing visceral fat accumulation has become a strategy to improve MHO health. Exercise intervention in people with abdominal obesity can not only reduce the amount of visceral fat but also increase the amount of skeletal muscle. Exercise can increase the secretion of IL-6 in skeletal muscle, which can stimulate fat decomposition (Wedell-Neergaard et al., 2019) and promote the increase of circulating lipids (Stanford et al., 2018). When motion changes, PPAR *γ* and PGC1-α expressions increase the body’s energy consumption (Boström et al., 2012). Cyclic lipids can be used as an important energy material in the energy supply, and the effective utilization of lipids is the premise to reduce the amount of fat. Energy consumption takes place mostly in skeletal muscle. Exercise can increase lipid consumption by activating the skeletal muscle G protein-coupled receptor Gpr35 (Agudelo et al., 2018). Exercise appears to promote transesterification and increase the absorption and utilization of fatty acids by skeletal muscle (Stanford et al., 2018). In addition, exercise can secrete specific proteins through actin to brown white fat. Brown fat can increase energy consumption and resist inflammation (Kong et al., 2018). These evidences suggest that exercise can improve skeletal muscle’ ability to consume and utilize fatty acids, secrete specific proteins, affect adipose tissue, and finally reduce the amount of visceral fat, hence reducing the risk of MHO disease.

### Exercise Increases the Level of Adiponectin and Improves the Metabolic State of Metabolically Healthy Obesity

**FIGURE 2:**
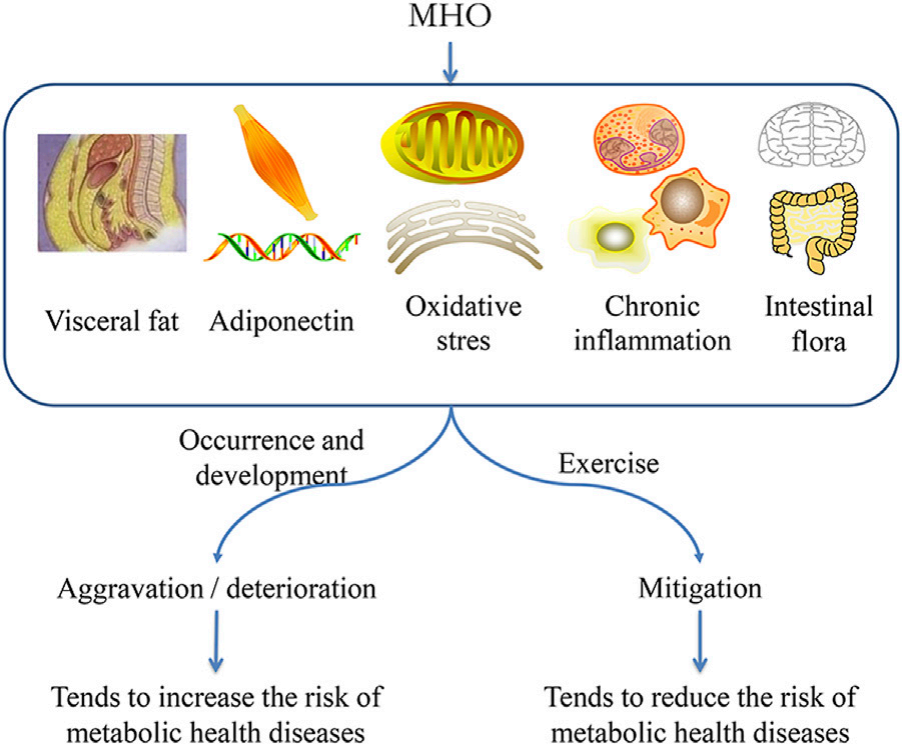
Ways to reduce the health harm of MHO by exercise.

Adiponectin is induced by proinflammatory mediators (such as lipopolysaccharide), cytokines, and a high-fat diet. It can not only regulate metabolism and affect fat distribution (Justice et al., 2019), but it also has a variety of protective effects on obesity-related cardiovascular metabolic disorders (Hui et al., 2015). Adiponectin can act on actin in an autocrine/paracrine manner, and the subsequent biological effects of actin provide a protective mechanism against metabolic damage (Martinez-Huenchullan et al., 2020). If the content of MHO adiponectin is low, the above protective reactions will be maladjusted, promoting the development of metabolic injury. Exercise can increase the concentration of plasma adiponectin (Sirico et al., 2018) or prevent or even reverse the dysregulation process (Figure 2). The pathway of adiponectin concentration on metabolic health is related to adiponectin signal transduction (Mao et al., 2006). When adiponectin binds to adiponectin receptor 1 and receptor 2, it induces extracellular Ca2+ influx and then activates AMPK/SIRT1, which regulates the expression of PGC-1 alpha and improves mitochondrial function (Iwabu et al., 2010). It can also increase glucose uptake, promote membrane translocation of glucose transporter 4 (GLUT4), and promote lipid oxidation. The changes in a series of receptor downstream events triggered by the increase of adiponectin concentration generated by exercise can reduce the severity of micro metabolic disorder of MHO and its health risks.

### Exercise Reduces Oxidative Stress and Improves the Metabolic State of Metabolically Healthy Obesity

According to the analysis results previously mentioned, the change of oxidative stress level is one of the manifestations of MHO’s micro metabolic changes, so improving its oxidative stress level may be one method to improve its metabolic health. The free radicals leading to oxidative stress are mainly produced in mitochondria. Obesity and malnutrition can cause imbalance of mitochondrial biosynthesis, increase mitochondrial fragmentation, and increase oxidative stress in skeletal muscle (Chen et al., 2018). This leads to mitochondrial dysfunction (decrease of O_2_ respiration and increase of oxidative stress in skeletal muscle), which reduces MHO lipid metabolism (Heo et al., 2017), triggers apoptosis and autophagy (Gao, 2019), increases the risk of metabolic diseases (Heo et al., 2017). Therefore, improving the oxidative stress level of MHO is of great significance.

Exercise can improve the level of oxidative stress in children (Rosa et al., 2011) and adults with obesity (Oh et al., 2013). Experimental intervention studies demonstrate that 4-weeks exercise can improve the activities of antioxidant enzymes such as superoxide dismutase and glutathione peroxidase (GPX) in obese adolescents (Li et al., 2017). In addition, exercise can improve the expression of mitochondrial proteins such as PGC1 alpha, p53, and AMPK (thr172) (Mendham et al., 2016), hence reducing the level of oxidative stress. Exercise can maintain the balance between mitochondrial dynamics and mitosis, reduce apoptotic signal transduction in obese skeletal muscle, and reduce mitochondrial dysfunction (Heo et al., 2017; Chen et al., 2018). Therefore, exercise can improve mitochondrial function and reduce the level of oxidative stress by enhancing antioxidant enzyme activity, improving mitochondrial protein expression, and optimizing mitochondrial dynamics, hence improving MHO’s metabolic state and improving health.

### Exercise Reduces Chronic Inflammation and Improves the Metabolic State of Metabolically Healthy Obesity

According to the theory of chronic inflammation of obesity, obesity is said to be characterized by chronic inflammation (Yudkin et al., 1999). The risk of obesity is associated with chronic inflammation (Yu et al., 2019). MHO is a subtype of obese people. It resembles obesity and cannot escape the invasion of chronic inflammation. Chronic inflammation is also a manifestation of metabolic imbalance at the microlevel of MHO. Based on the principle of healthy metabolic balance, the means to improve MHO chronic inflammation can improve its metabolic health level (Kotas and Medzhitov, 2015), in which exercise is a safe and effective strategy.

Regular exercise has been proven to lower CRP levels in both adults (Trachta et al., 2014) and children (Li et al., 2010). Other studies have found that exercise has no significant effect on CRP content in obese people (Wong et al., 2008). The physical activity level of obese people was related to inflammatory markers such as sTNF-R1, sTNF-R2, IL-6, and CRP, according to cross-sectional survey data using large samples and multiple indicators (Pedrinolla et al., 2018). It is verified that exercise can reduce the body’s inflammatory levels.

Research shows that exercise lowers visceral fat, reduces proinflammatory cytokine secretion, and reduces inflammation level. Exercise can improve endoplasmic reticulum stress, reduce inflammatory response, reduce oxidative stress reducing unfolded protein response, and then inhibit the inflammatory pathway mediated by inducible nitric oxide synthase (iNOS) (Yang et al., 2015). Exercise can also increase fatty acid oxidation in obese people (Krause et al., 2019) and reduce the inflammatory response triggered by saturated long-chain fatty acids (Lancaster et al., 2018). The improvement of chronic inflammation of MHO is the performance of health promotion. Exercise can reduce the degree of proinflammatory response and improve the health level of MHO through methods mentioned above.

### Effect of Exercise on Metabolically Healthy Obesity Omics

According to relevant proteomic studies, there are considerable differences in the expression of a variety of proteins between the obese group and the healthy control group. These proteins are mainly related to the tricarboxylic acid cycle, gluconeogenesis, and muscle contraction. Their changes could be a major factor in the development of abnormal obesity metabolism. Exercise can affect the expression of a variety of energy metabolism-related proteins (ECH1, HBA, MLRS, MLRV, MYH1, MYH2, PYGM, G3P, NDUS2, ATP5F1, ATPB, COX2, and APOA1) in obese group. The changes of these proteins may be the potential mechanism for improving MHO health (Srisawat et al., 2017). The metabonomics study of obesity found that the severity of obesity is related to reduce serine and glycine concentrations. Lower concentrations of serine and glycine may affect sphingolipid metabolism. Increasing exercise in obese people can increase the concentrations of serine and glycine, thus improving sphingolipid metabolism and reducing health risks (Palmnäs et al., 2018). The metabonomics study of obesity conducted in our laboratory shows that exercise can improve fatty acid decomposition and sphingolipid metabolism of overweight or obese people, increase glucuronidation, and promote the discharge of metabolic waste and toxins. Exercise can also improve the distribution of intestinal flora (Mayengbam et al., 2019), promote the transformation and utilization of energy substances by microorganisms, and reduce the production of harmful metabolites (Wernstedt Asterholm et al., 2014; Laurans et al., 2018). These omics studies reveal that exercise can help to lower the health risks associated with obesity by improving MHO energy metabolism-related proteins, optimizing lipid metabolism pathway, and increasing metabolic waste and toxin excretion.

In conclusion, the micro metabolic disorder of MHO is the reason for the increase of disease risk. Using the micro disorder pathway as the target for intervention can help to mitigate the health risks of MHO. Exercise can improve metabolic activity, increase health benefits, and reduce disease risk by correcting the micro disorder of MHO (Figure 2).

## Summary

MHO demonstrated abnormal metabolism in fat distribution, adiponectin pathway, chronic inflammation, oxidative stress, and various omics at the microlevel, despite having normal metabolism in the criteria of metabolic syndrome and insulin resistance. In view of this phenomenon, this review proposes that MHO is in the state of micro metabolic disorder. This viewpoint explains why, in cross-sectional studies, the disease risk of MHO is higher than that of people with normal metabolism and lower than that of obesity with abnormal metabolism. It can also explain why prospective studies conclude that MHO has a higher risk of disease in the future, which is also the basis for the selection of intervention targets of health promotion strategies. The micro disorder of MHO metabolism is the initial reason for the increase of metabolic diseases in the future.

Exercise reduces visceral fat, increases the level of adiponectin, reduces oxidative stress, reduces chronic inflammation, correct the abnormal micro metabolic pathway of MHO, regulate metabolic homeostasis, and improve metabolite activity. It is not only a supplement or possible alternative to the traditional healthcare prevention/treatment strategy, but it is also an important strategy to reduce the health risks of MHO.

Microlevel research will help us to understand the metabolic characteristics of MHO, and different micro metabolic characteristics may develop into different diseases. For example, MHO can develop into type 2 diabetes, hyperlipidemia, coronary heart disease, and other diseases, although it is microscopic mechanism is unknown. Therefore, screening different micro indicators to predict the specific disease types and mechanisms of MHO is of great significance for targeted preventive measures. Exercise can improve MHO micro metabolic disorder in a variety of ways, but many monitoring indicators may only improve the accompanying effect of metabolic health. The pathways, genes, proteins, and metabolites that play a key role and/or have high specificity are not well understood. The mastery of these biological data will be critical in the treatment of MHO micro metabolic disorder and the reduction of disease risk.
